# Genomic Insights of a Methicillin-Resistant Biofilm-Producing *Staphylococcus aureus* Strain Isolated From Food Handlers

**DOI:** 10.1155/2024/5516117

**Published:** 2024-07-20

**Authors:** Fatimah Muhammad Ballah, M. Nazmul Hoque, Md. Saiful Islam, Golam Mahbub Faisal, Al-Muksit Mohammad Taufiquer Rahman, Mst. Minara Khatun, Marzia Rahman, Jayedul Hassan, Md. Tanvir Rahman

**Affiliations:** ^1^ Department of Microbiology and Hygiene Faculty of Veterinary Science Bangladesh Agricultural University, Mymensingh 2202, Bangladesh; ^2^ Molecular Biology and Bioinformatics Laboratory Department of Gynaecology Obstetrics and Reproductive Health Bangabandhu Sheikh Mujibur Rahman Agricultural University, Gazipur 1706, Bangladesh; ^3^ Department of Animal Sciences University of California-Davis, Davis, California CA 95616, USA; ^4^ Department of Medicine Rajshahi Medical College, Rajshahi 6100, Bangladesh

**Keywords:** antibiotic resistance, biofilm production, food handlers, hand swab, *Staphylococcus aureus*, virulence

## Abstract

Methicillin-resistant *Staphylococcus aureus* (MRSA) is an important zoonotic pathogen associated with a wide range of infections in humans and animals. Thus, the emergence of MRSA clones poses an important threat to human and animal health. This study is aimed at elucidating the genomics insights of a strong biofilm-producing and multidrug-resistant (MDR) *S. aureus* MTR_BAU_H1 strain through whole-genome sequencing (WGS). The *S. aureus* MTR_BAU_H1 strain was isolated from food handlers' hand swabs in Bangladesh and phenotypically assessed for antimicrobial susceptibility and biofilm production assays. The isolate was further undergone to high throughput WGS and analysed using different bioinformatics tools to elucidate the genetic diversity, molecular epidemiology, sequence type (ST), antimicrobial resistance, and virulence gene distribution. Phenotypic analyses revealed that the *S. aureus* MTR_BAU_H1 strain is a strong biofilm-former and carries both antimicrobial resistance (e.g., methicillin resistance; *mec*A, beta-lactam resistance; *bla*Z and tetracycline resistance; *tet*C) and virulence (e.g., *sea*, *tsst*, and *PVL*) genes. The genome of the *S. aureus* MTR_BAU_H1 belonged to ST1930 that possessed three plasmid replicons (e.g., rep16, rep7c, and rep19), seven prophages, and two clustered regularly interspaced short palindromic repeat (CRISPR) arrays of varying sizes. Phylogenetic analysis showed a close evolutionary relationship between the MTR_BAU_H1 genome and other MRSA clones of diverse hosts and demographics. The MTR_BAU_H1 genome harbours 42 antimicrobial resistance genes (ARGs), 128 virulence genes, and 273 SEED subsystems coding for the metabolism of amino acids, carbohydrates, proteins, cofactors, vitamins, minerals, and lipids. This is the first-ever WGS-based study of a strong biofilm-producing and MDR *S. aureus* strain isolated from human hand swabs in Bangladesh that unveils new information on the resistomes (ARGs and correlated mechanisms) and virulence potentials that might be linked to staphylococcal pathogenesis in both humans and animals.

## 1. Introduction

*Staphylococcus aureus*, an opportunistic and notorious zoonotic pathogen, is responsible for a wide range of infections in humans and animals [1, 2]. *S. aureus* is notable for its ability to colonize a wide range of vertebrate hosts, each representing a distinct ecological niche [3–5]. *S. aureus* is a known commensal of both man and animals. The bacterium could invade the skin, mucous membranes, and internal organs, causing severe illness in animals and humans. In humans, it is responsible for severe nosocomial and community-associated infections. Human diseases associated with *S. aureus* include osteomyelitis, pneumonia, meningitis, arthritis, endocarditis, septicaemia, deep tissue abscesses, and skin and soft-tissue infections (SSTIs), as well as toxic shock syndrome, among others [6, 7]. *S. aureus* is also a common cause of wound and urinary tract infections [8]. On the other hand, *S. aureus* infections are most commonly reported as a cause of mastitis in dairy animals [9, 10], osteomyelitis in poultry, and skin abscesses, mastitis, and septicemia in farmed rabbits [1, 11]. The pathogenicity of *S. aureus* is triggered by a number of characteristics, such as invasive components, toxin-associated virulence factors, biofilm formation, and antibiotic resistance [6, 12]. These characteristics also assist these organisms in becoming more resistant to hostile environments, developing infections, and escaping the immune system of the host.

Animal-associated *S. aureus* is also known to carry diverse antimicrobial resistance genes (ARGS) and can therefore act as a reservoir for transmitting resistant strains to humans [6]. In recent 20 years, *S. aureus* infections have become more dangerous and costly to treat because of increasing prevalence of antimicrobial resistance in *S. aureus* due to the widespread use of antibiotics [13]. Notably, the emergence and spread of MDR and MRSA (methicillin-resistant *S. aureus*) are not limited to hospital and community settings but also pose an important threat in veterinary medicine, agricultural systems, and food production [6, 14, 15]. The ability to switch and adapt to new hosts is a dynamic and constant feature of the evolution of *S. aureus* [6].

Recently, MRSA continues to be a significant pathogen in both hospital and community settings [6]. MRSA consists of virulent zoonotic strains of *S. aureus* that are specifically characterized by their resistance to cefoxitin and methicillin. *S. aureus* develops resistance to methicillin, as seen in MRSA, through the acquisition of the *mecA*, *mecB*, or *mecC* genes [1, 16]. MRSA can be clinically determined through polymerase chain reaction (PCR)-based detection of the *mecA*, *mecB*, or *mecC* genes, as well as by assessing resistance to cefoxitin [1, 17]. The *mec*A gene mainly encodes for the penicillin-binding protein responsible for this kind of antimicrobial resistance. MRSA consistently exhibits a MDR pattern, not only to penicillin but also to various classes of antimicrobials, including macrolides, fluoroquinolones, aminoglycosides, tetracyclines, and lincosamides [10, 16, 18]. MRSA can induce severe infectious diseases in humans, and the emergence of MDR virulent MRSA strains is a significant public health problem [19, 20]. MRSA is a major pathogen in chronic infections due to its biofilm-forming ability, a key virulence factor aiding its persistence in the environment and host. Biofilm production in *S. aureus* is often linked to polysaccharide intracellular adhesin (PIA) synthesis, encoded by the *ica* operon [2, 21]. Therefore, MRSA is considered a historic emergent zoonotic pathogen with public health and veterinary importance [18]. However, the mechanisms underlying the biofilm formation of MRSA strains from different types of human infections are not fully understood. Therefore, understanding the genomic characteristics of biofilm-producing MRSA in other hosts would be key to advancing a One Health approach to attain optimal health for people, domestic animals, wildlife, plants, and the environment.

Genomic data of biofilm-producing MRSA strains will support molecular epidemiology for the surveillance of outbreaks and has the potential for future genotypic antimicrobial susceptibility testing as well as the identification of novel therapeutic targets and prognostic markers. In this article, we describe the epidemiological distribution, genomic diversities, ARGs, and virulence factors/genes found in a biofilm-forming MDR *S. aureus* MTR_BAU_H1 strain isolated from the food handlers' hand swab samples in Bangladesh. To elucidate the genetic diversity, molecular epidemiology, sequence type (ST), and distribution of antimicrobial resistance and virulence genes in *S. aureus* MTR_BAU_H1, high throughput whole-genome sequencing (WGS) and downstream bioinformatic analysis were performed.

## 2. Materials and Methods

### 2.1. Isolation and Identification of MRSA

In this study, we used the *S. aureus* strain MTR_BAU_H1, which is a type of MRSA found in hand swabs of food handlers in Bangladesh [4, 22]. The isolate was stored at −80°C in a stock medium containing LB (Luria-Bertani) broth (HiMedia, India) and 20% glycerol. In in vitro antibiogram assays conducted with the disk diffusion method, following the guidelines outlined in the Clinical Laboratory Standards Institute (CLSI) M100 33rd Edition [23], the MTR_BAU_H1 isolate exhibited resistance to both methicillin and cefoxitin, confirming its MRSA phenotype. Subsequently, we confirmed the MTR_BAU_H1 strain as MRSA by conducting a specific PCR assay targeting the methicillin resistance gene (*mec*A) [22]. The isolate was subcultured on MSA (Mannitol Salt Agar) (HiMedia, India) media. At first, one loopful of overnight-cultured nutrient broth was streaked on separate MSA plates and incubated aerobically at 37°C for 24 h. Isolates having golden-yellow colonies on MSA agar plates were assumed to be *S. aureus*. Thereafter, purity of the colonies was checked by culturing on 5% bovine blood agar (HiMedia, India) plates at 37°C for 24 h [4, 9].

### 2.2. Genomic DNA Extraction and WGS of an MRSA Strain

Genomic DNA from *S. aureus* MTR_BAU_H1 was extracted from an overnight nutrient broth culture using QIAamp DNA Mini Kit (QIAGEN, Hilden, Germany), and the quantity was checked with a NanoDrop 2000 UV-Vis Spectrophotometer (Thermo Fisher, Waltham, MA, USA). For short-read WGS, the Nextera™ DNA Flex Library Prep Kit (Illumina, San Diego, USA) was used to generate libraries from 1 ng DNA. Libraries were cleaned (AMPure XP beads; Beckman Coulter) and sequenced using Illumina MiSeq sequencer using a 2 × 250 paired-end protocol. Reads were trimmed using Trimmomatic v0.39 [24] and quality checked using FastQC v0.11.7 (Babraham Bioinformatics).

### 2.3. Assembly and Annotation of the WGS Data

We conducted reference-guided assembly using SPAdes v. 3.12.1 [25] on reads with a phred score > 20. The quality of the resulting assembly was assessed utilizing QUAST v.5.0.2 [26]. The annotation of the draft assembly of MTR_BAU_H1 was performed using the National Center for Biotechnology Information (NCBI) Prokaryotic Genomes Annotation Pipeline (PGAP) (https://www.ncbi.nlm.nih.gov/genome/annotation_prok/). Genome completeness analysis was carried out with BUSCO (Benchmarking Universal Single-Copy Orthologs) v.4.1.2 with “bacteria_'odb10” data set [27].

### 2.4. Phylogenetic Analysis, Genomic Comparison, and Sequence Typing

The MTR_BAU_H1 genome and the GenBank reference sequence genomes (*n* = 26) of relevant MRSA clones were used in phylogenetic analysis. We selected MRSA genomes for phylogenetic analysis, prioritizing closely related strains with high-quality and complete genome assemblies, including associated metadata to contextualize the analysis, and validating selections to ensure accuracy and reliability. Genomes were submitted to alignment by the MAFFT version 7 (https://mafft.cbrc.jp/alignment/server/) algorithm using default parameters [28, 29]. Orthologs were extracted from the resulting .xmfa file using stripSubsetLCBs (http://darlinglab.org/mauve/snapshots/2015/2015-01-09/linux-x64/) with the minimum locally colinear block length set to 500 bp and the number of genomes set to 75. The result was then converted to FASTA format using the xmfa2fasta.pl script, and the FASTA-formatted alignment was then converted to phylip format using the fasta2phylip.pl script (both parts of the BioPerl package) (http://www.bioperl.org/). The phylogenetic tree (phylip format) was subsequently imported to iTOL (v. 3.5.4) (http://itol.embl.de/) [30] for better visualization, and bootstrap values were reported for each branch.

The circular visualization of the *S. aureus* MTR_BAU_H1 genome was performed using CGViewer [31]. Multiple genome (*n* = 4) alignments and visualizations were performed using BLAST Ring Image Generator (BRIG) version 0.95 with *S. aureus* KUN1163 (GenBank Accession No.: AP020324.1) as reference, one of the important MRSA genomes. Plasmid replicons for the genome sequences were predicted by using PlasmidFinder (https://cge.cbs.dtu.dk/services/PlasmidFinder/) with the setting of the threshold for a minimum of 95% identity over 60% coverage of length [28, 32]. The CRISPRimmunity (http://www.microbiome-bigdata.com/CRISPRimmunity/index/home), a clustered regularly interspaced short palindromic repeat (CRISPR)-oriented web tool, was used to assess the CRISPR arrays and associated genes in the study genome. Phage-associated genes and genomic regions in the MTR_BAU_H1 genome were identified using PHAge Search Tool Enhanced Release (PHASTER) server (http://phaster.ca/) [33]. The completeness of the predicted prophage-associated regions was defined by three scenarios according to how many genes/proteins of a known phage the region contained: intact (≥ 90%), questionable (90%–60%), and incomplete (≤ 60%). The core genome (cg) multilocus sequence typing (cgMLST) was performed by using the *S. aureus* database in the BacWGSTdb 2.0 web server [34].

### 2.5. Prediction of Antimicrobial Resistance and Virulence Genes

The ABRicate v1.0.1 (https://github.com/tseemann/abricate) bundled with multiple databases—NCBI AMRFinder [35], CARD 2020 [36], ARG-ANNOT [37], ResFinder 4.0 [38], and MEGARes 2.0 [39]—was used to predict ARGs in the assembled genome. The ARG selection criteria were set to perfect (100% identity) and strict (> 95% identity) hits only to the curated reference sequences in the databases. Bioinformatics and Evolutionary Genomics (https://bioinformatics.psb.ugent.be/webtools/Venn/) tool was used to draw the Venn diagrams for the detected ARGs. The virulence genes were identified in the de novo assembled contigs of the *S. aureus* MTR_BAU_H1 genome using the web-service VirulenceFinder 2.0 (https://cge.cbs.dtu.dk/services/VirulenceFinder/) and VFanalyzer of virulence factor database (VFDB, http://www.mgc.ac.cn/VFs/) [40]. The virulence genes in the MTR_BAU_H1 genome were predicted using 90% nucleotide identity and query coverage [22, 41]. For VirulenceFinder 2.0, identification thresholds were set at 90% over a minimum identity length of 60% [42, 43].

### 2.6. Genomic Functional Potential Analysis

The draft genome of the *S. aureus* MTR_BAU_H1 strain was annotated using the RAST (Rapid Annotation using Subsystem Technology) server, version 2.0 [44], and KEGG (Kyoto Encyclopaedia of Genes and Genomes) automatic annotation server (KAAS) [45]. The RAST server provided data on the distribution of genes in various subsystem categories [46].

## 3. Results

### 3.1. Phenotypic and Genotypic Features of the *S. aureus* Strain MTR_BAU_H1

We previously extensively studied the phenotypic traits of the *S. aureus* strain MTR_BAU_H1 [1, 16]. The MTR_BAU_H1 strain was identified as a strong biofilm producer that harboured four biofilm-associated genes such as *icaA*, *icaB*, *icaC*, and *icaD*. In vitro antimicrobial susceptibility test (AST) revealed that the *S. aureus* MTR_BAU_H1 strain was phenotypically resistant to azithromycin, chloramphenicol, tetracycline, oxacillin, ampicillin, penicillin, and cefoxitin. However, this strain remained susceptible against gentamicin, ciprofloxacin, erythromycin, and cotrimoxazole. Therefore, *S. aureus* MTR_BAU_H1 was considered as a MDR strain with predominantly higher resistance against oxacillin (100.0%), ampicillin (100.0%), penicillin (100.0%), cefoxitin (100.0%), and erythromycin (55.0%). Gene-specific PCR confirmed that this MDR strain possessed three ARGs conferring resistance to methicillin (*mec*A), beta-lactams (*bla*Z), and tetracycline (*tet*C), and three enterotoxin-producing virulence genes such as *sea*, *tsst*, and *PVL*.

### 3.2. Genome Characteristics of the *S. aureus* Strain MTR_BAU_H1

**Figure 1:**
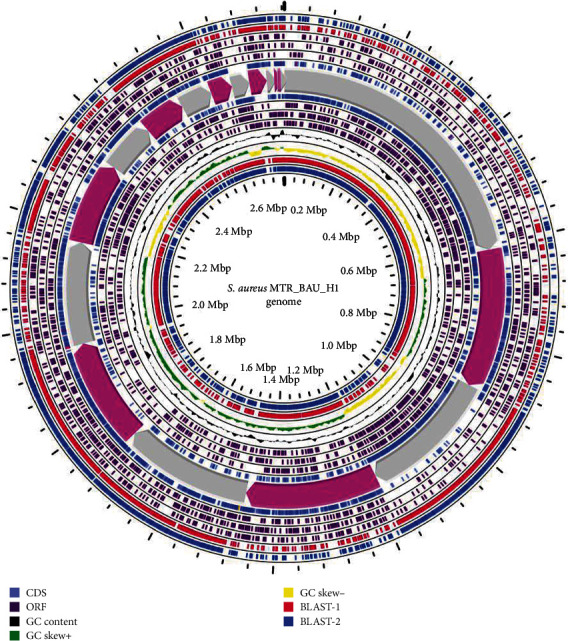
Circular genome representation of *Staphylococcus aureus* MTR_BAU_H1 genome compared with BLAST-1: *S. aureus* strain CC1153-MRSA (GenBank accession: CP065857.1) and BLAST-2: *S. aureus* strain WBG8287 (GenBank accession: CP070986.1). The outermost ring: BLAST-2 results for *S. aureus* strain WBG8287 (blue) followed by BLAST-1 results for *S. aureus* strain CC1153-MRSA (red) representing the positions covered by the BLAST comparative alignment results (BLASTN), coding sequences (CDS) (lavender blue color), open reading frame (ORF) (indigo color), G + C content (black), G + C positive skew (green), and G + C negative skew (yellow). Image created using CGview Server.

The general genomic features of the *S. aureus* strain MTR_BAU_H1, used for WGS, are summarized in Table 1. The size of the MTR_BAU_H1 draft genome is approximately 2.76 Mb (32.6% GC content), of which 2,764,427 bp belongs to the genomic region that corresponds to the chromosome of *S. aureus* (GCA_026242155.1), with 50× genome coverage and 100% genome completeness. The predicted coding sequences (CDSs), tRNAs, and rRNAs were 2698, 46, and 2, respectively. The final assembly contained 21 contigs with N50 of 279,348 bp length. The largest contig assembled was 609,991 bp in length (Table 1). A physical genome map of *S. aureus* MTR_BAU_H1 genome in comparison to two other MRSA reference strains *S. aureus* strain CC1153-MRSA (BLAST 1; GenBank accession: CP065857.1) and *S. aureus* strain WBG8287 (BLAST 2; GenBank accession: CP070986.1) is shown in Figure 1. The MTR_BAU_H1 genome possessed two CRISPR arrays (in contig 5) with 11 gene signatures such as *cas*1, *cas*2, *cas*6, *cas*10, *csm*2gr11, *csm*3gr7, *csm*4gr5, *csm*5gr7, *csm*6, RT, and *Tns*C. The *cas* genes were found adjacent to both CRISPR arrays (Figure S1). The MTR_BAU_H1 genome also harboured seven prophages (three in contig 1, two in contig 7, and one in contig 3 and 8) and three plasmid replicons (e.g., rep16, rep7c, and rep19), homologous to previously reported elements (Table 1). The predicted rep16 (accession no.: CP000737), rep7c (accession no.: BX571857), and rep19 (accession no.: AB699881) plasmid replicons were 744, 821, and 984 bp in lengths, respectively. Among these plasmids, rep16 and rep19 were predicted in contig 15 (position: 16940–17983 and 18109–19093, respectively) while rep7c plasmid was identified in contig 5 (position: 90955–91775) of the MTR_BAU_H1 genome.

### 3.3. Genomic Comparison Revealed Genetic Resemblance Between the MTR_BAU_H1 Strain and MRSA Clones

**Figure 2:**
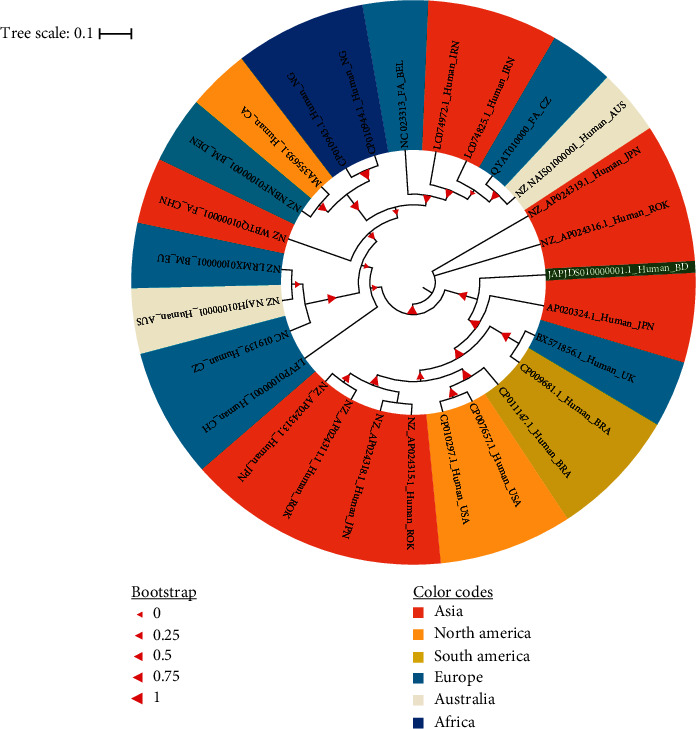
The evolutionary relationships of *Staphylococcus aureus* genomes sequenced from six continents of the world. Whole genome sequences of 26 strains of human and animal origin retrieved from NCBI were used for phylogenetic analysis. The midpoint rooted tree was constructed using the NCBI Tree Viewer (https://www.ncbi.nlm.nih.gov/tools/treeviewer/) and visualized with iTOL (interactive tree of life). The evolutionary relationship was inferred using the maximum likelihood method. Different colors (e.g., pinkish orange for Asia, yellowish orange for North America, citrine for South America, pacific blue for Europe, merino for Australia, and cyan blue for Africa) are assigned according to the close evolutionary relatedness (clade) of the genomes. The scale bar is in the unit of the number of substitutions per site. The values on the branches are bootstrap support values based on 1000 replications. All the sequences were indicated by their accession numbers followed by the host and country code. The country codes according to the standard abbreviation are as follows: United States of America (USA), United Kingdom (UK), Japan (JPN), Republic of Korea (ROK), Brazil (BRA), Czech Republic (CZ), Switzerland (CH), China (CHN), Australia (AUS), Denmark (DEN), Belgium (BEL), Iran (IRN), Canada (CA), Nigeria (NG), European Union (EU), and Bangladesh (BD). The Bangladeshi MTR_BAU_H1 genome is highlighted in dark green.

**Figure 3:**
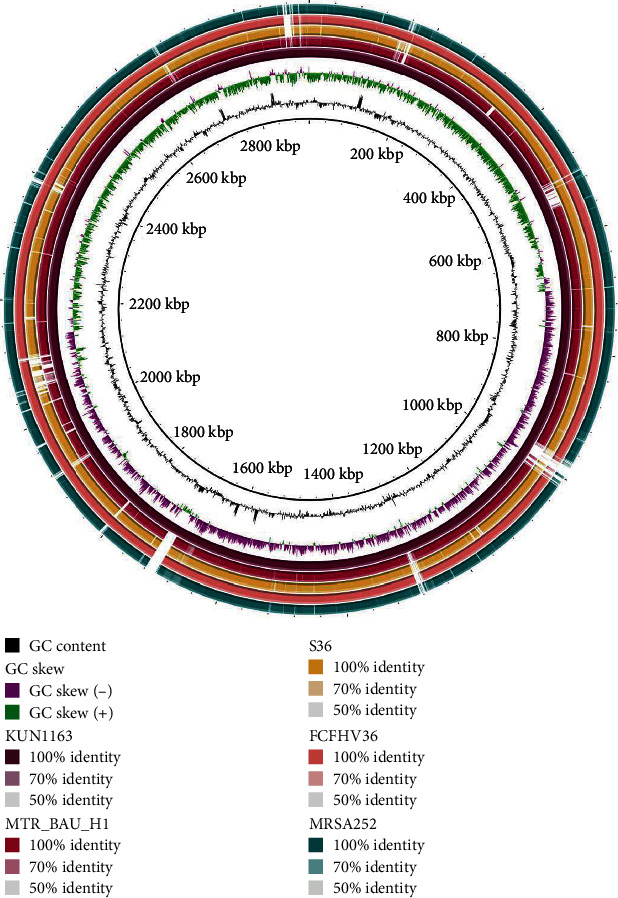
Circular representation of the *Staphylococcus aureus* complete genomes. Circles (from inside to outside) 1 and 2 (GC content: black line and GC skew: purple (− ve) and green (+ ve) lines); circle 3 (*S. aureus* strain KUN1163; velvet circle); circle 4 (mapped *S. aureus* strain MTR_BAU_H1 genome with *S. aureus* KUN1163 genome; pinkish red circle); circle 5 (mapped *S. aureus* strain S36 genome with *S. aureus* KUN1163 genome; yellowish orange circle); circle 6 (mapped *S. aureus* strain FCFHV36 genome with *S. aureus* KUN1163 genome; light coral circle); and circle 7 (mapped *S. aureus* strain MRSA252 genome with *S. aureus* KUN1163 genome; greenish blue circle). BRIG 0.95 was used to build the circular representation. Mapping studies were done using BLASTn with an *e*-value cut-off 1*e*-5.

The maximum likelihood phylogenetic tree based on the alignment of 26 whole genome sequences of human and animal origin retrieved from the GenBank database of the NCBI showed a great genetic relatedness between the study genome (JAPJDS010000001.1_Human_BD; GenBank: JAPJDS010000001.1) and the retrieved reference genomes of MRSA clones (Figure 2). This phylogenetic inference revealed that the human strain *S. aureus* MTR_BAU_H1 grouped into a clade, which includes genomes of human-derived MRSA strains. This clade grouped mostly with the genomes of strains from the Asian, American, and European continents (e.g., Japan, Republic of Korea, Brazil, the United States, and the United Kingdom). Based on the phylogenetic analysis, the completely closed genome of the human strain *S. aureus* MTR_BAU_H1 is another Japanese strain of *S. aureus* (GenBank accession: AP020324.1) isolated and sequenced from a human patient with severe disease (Figure 2). In addition, a comparative genomic analysis was performed among the strains where *S. aureus* KUN1163 genome (GenBank accession: GCA_008619075.1) was used as a reference. The analysis of the circular genomes by BRIG (Figure 3) confirmed the high nucleotide conservation. The genomic map obtained from the BRIG comparison did not show large-scale variation between the bacterial genome sequences, and a significant number of nonhomologous regions were found around the reference genome with over 95% identity. The MTR_BAU_H1 strain was seen sharing more common regions of genetic variation with the MRSA clones (e.g., S36, FCFHV36, and MRSA252) than with the reference MRSA strain (e.g., KUN1163) at different sites on the genomes (Figure 3). Most of these nonhomologous regions might be linked to transposable elements.

**Figure 4:**
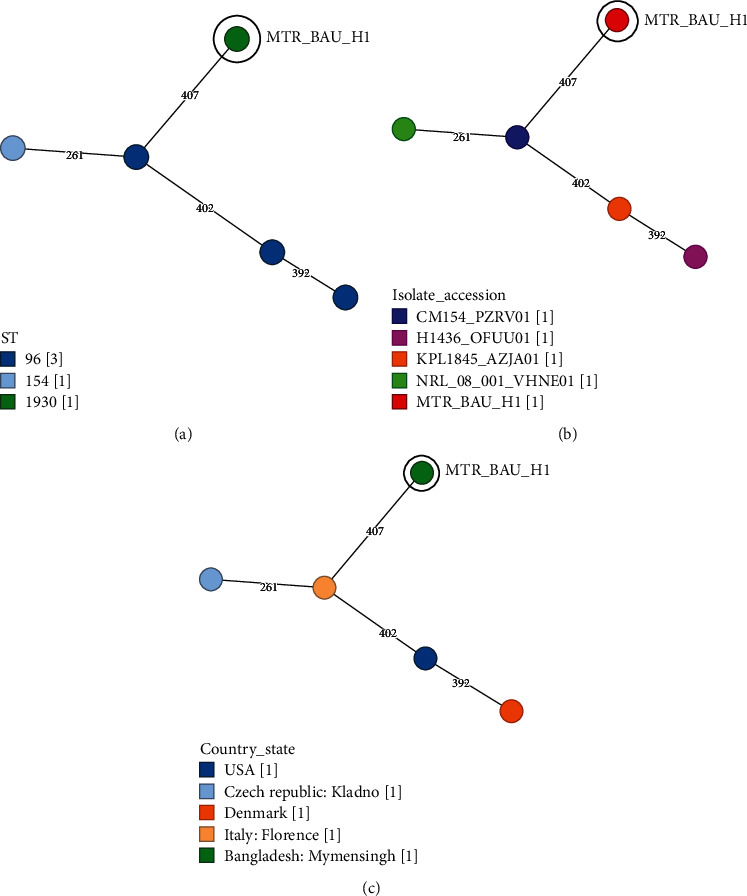
Evolutionary phylogenetic relationship between *S. aureus* strain MTR_BAU_H1 and three *S. aureus* strains belonging to ST96 and one *S. aureus* strain belonging to ST154 currently available in the NCBI GenBank database by core genome multilocus sequence typing (cgMLST) analysis. (a) Phylogenetic relationship based on sequence type (ST), (b) isolate accession numbers, and (c) origin of the isolates. Different color codes represent different (a) sequence types (STs), assigned closely related (b) isolates, and their (c) originating country or state. The black circles indicate ST1930 S*. aureus* strain MTR_BAU_H1 originated from Bangladesh.

To assess the genetic relatedness between the *S. aureus* MTR_BAU_H1 isolate and four related *S. aureus* strains based on seven-gene MLST (*arc*C, *aro*E, *glp*F, *gmk*, *pta*, *tpi*, and *yqi*L) on cgMLST scheme, we visualized the number of allelic differences of the isolates (Figure 4). According to the cgMLST scheme, the *S. aureus* MTR_BAU_H1 strain belongs to ST1930 which differed by 407 cgMLST alleles. The cgMLST analysis revealed that the closest relatives of MTR_BAU_H1 are three ST96 and one ST154 strains of *S. aureus* originating from the swab and blood samples of clinically infected people of the United States, Czech Republic, Denmark, and Italy. Therefore, four cgMLST clusters of genetically distinguishable isolates were observed (Figure 4). These *S. aureus* strains (ST96 and ST154) also harboured different ARGs including *bla*Z, *mec*A, and *tet*(M).

### 3.4. Resistome Assessment in *S. aureus* Strain MTR_BAU_H1

**Figure 5:**
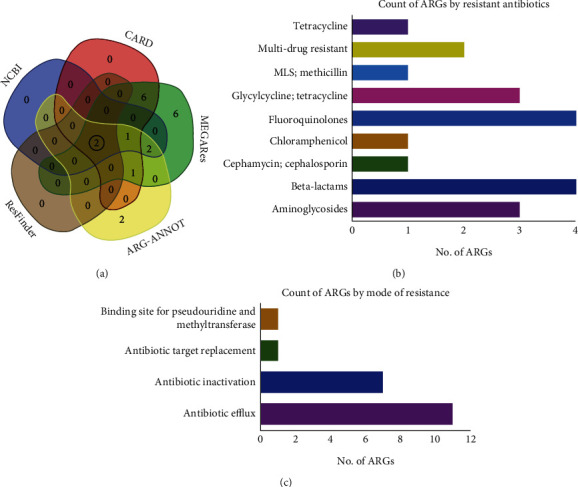
An overview of the antimicrobial resistance genes (ARGs), resistant antibiotics, and resistance mechanisms associated with the genome of MTR_BAU_H1. (a) Venn diagram showing unique and shared antibiotic-resistant genes (ARGs) detected through CARD, MEGARes, ARG-ANNOT, ResFinder, and NCBI databases. (b) Count of ARGs against different antibiotics. (c) ARGs count according to different mechanisms of resistance. Two ARGs such as *mecA* and *blaZ* were found to be shared in all of the five databases used for annotation of the genome, and these shared ARGs are highlighted in black circle.

A total of 42 ARGs, including *mec*A, *bla*Z, and *tet*C belonging to 13 different groups, were identified in the genome analysis of MTR_BAU_H1 (Figure 5(a)). We further determined the number of ARGs associated with resistance to different antibiotics. The MTR_BAU_H1 was an MDR isolate harbouring ARGs resistant to *mec*A, *bla*Z, aminoglycosides, cephalosporin, chloramphenicol, fluoroquinolones, and tetracycline (Figure 5(b)). In addition, one macrolide-lincosamide-streptogramin (MLS) resistance gene (*rlm*H) and two MDR efflux pumps conferring genes *mgr*A and *lmr*S were identified and found to be resistant against MLS and lincomycin antibiotics. These ARGs represent a variety of resistance mechanisms such as antibiotic efflux mediated resistance, antibiotic inactivation, antibiotic target replacement, and binding site for pseudouridine and methyltransferase based on definitions in the CARD, MEGARes, ARG-ANNOT, ResFinder, and NCBI databases. However, among the detected ARGs, the genome of MTR_BAU_H1 carries the highest number (*n* = 11) of genes conferring antimicrobial resistance through antibiotic efflux (e.g., *tet*(38), *bla*I, *nor*A, *nor*B, *mgr*A, *arl*S, *arl*R, *mep*R, *mep*A, *lmr*S, and DHAP). Furthermore, seven genes (e.g., *bla*Z, *bla*R1, *bla*I, AAC(3), APH3-Prime, DHA-1, and apH-Stph) were identified as showing resistance through antibiotic inactivation mechanism (Figure 5(c)).

### 3.5. Virulence and Functional Characteristics of *S. aureus* Strain MTR_BAU_H1

**Figure 6:**
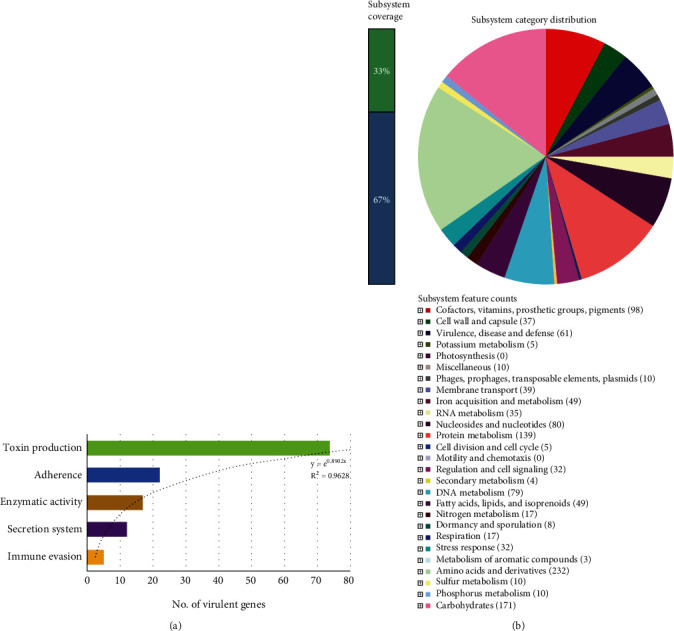
Genomic functional potentials of the *S. aureus* strain MTR_BAU_H1. (a) Virulence determinants of the genome. The *x*-axis represents the number of virulent genes while the *y*-axis shows different virulent mechanisms. (b) The SEED subsystem-based functional module distribution in *S. aureus* strain MTR_BAU_H1 genome. The whole genome sequence of the *S. aureus* strain MTR_BAU_H1 was annotated using the Rapid Annotation System Technology (RAST) server. The pie chart demonstrates the count of each subsystem feature and the subsystem coverage.

The genome sequence of the MTR_BAU_H1 isolate harbours 128 virulence genes (*n* = 128) deliberating virulence through five distinct classes of virulence mechanisms such as adherence, enzymatic activity, immune evasion, secretion system, and toxin production (Figure 6(a)). The predicted virulence genes showed > 91% nucleotide identity with the genes of the reference database (i.e., VFDB) (Table S1). Among the detected virulence genes, majority of the genes (57.81%) were found to be associated with toxin production including Panton–Valentine leukocidin (e.g., *luk*F-PV, *luk*S-PV), toxic shock syndrome (e.g., *tsst*), enterotoxin (e.g., *sea*, *seb*, *sec*, *sed*, *see*, *seg*, *she*, *sei*, and *sej*), and exfoliative toxin (e.g., *eta*, *etb*, *etc*, and *etd*). Moreover, 17.19%, 11.72%, 3.91%, and 9.38% genes were found to be correlated with adherence (e.g., *atl*, *ebh*, *clf*A-B, *cna*, *ebp*, *eap*/*map*, *efb*, *fnb*A-B, *ica*A-D, *ica*R, *sdr*C-H, and *spa*), enzymatic activity (e.g., *ssp*B-C, *hys*A, *geh*, *lip*, *ssp*A, *spl*A-F, *coa*, *sak*, and *nuc*), immune evasion (e.g., *ads*A, *chp*, *scn*, and *sbi*), and type VII secretion system (e.g., *esa*A, *esa*B, *esa*D, *esa*E, *esa*G, *ess*A-C, and *esx*A-D), respectively (Table S1).

The detected functional pathways were distributed under 273 SEED subsystems, which were represented mainly by the genes coding for metabolisms of amino acid and derivatives (18.83%); carbohydrates (13.88%); protein (11.28%); cofactors, vitamins, prosthetic groups, and pigments (7.95%); iron (3.98%); and fatty acids, lipids, and isoprenoids (3.98%) (Figure 6(b)). Results from the functional annotation revealed that 61 genes (4.95%) encoded for virulence, disease, and defense where 26 genes were annotated to be responsible for adhesion, 22 for resistance to antibiotics and toxic compounds, four for bacteriocins and ribosomally synthesized antibacterial peptides, and nine for invasion and intracellular resistance. In addition, genes related to membrane transport (3.17%), stress response (2.60%), and regulation and cell signaling (2.60%) were predicted in the *MTR_BAU_H1 genome*. The rest of the SEED subsystems had relatively lower abundances (< 2.0%) (Figure 6(b)). Investigating deeper into these SEED subsystem distributions, we found that genes coding for monosaccharide (25.2%) and central CHO (20.81%) metabolism, protein biosynthesis (74.26%), and lysine, threonine, methionine, and cysteine metabolism (20.62%) were top abundant metabolic functional categories in *S. aureus* MTR_BAU_H1. Moreover, oxidative stress (43.21%), type II protein secretion system (26.23%), chaperon pathway (12.74%), and folate and pterion metabolism (32.41%) were the predominating metabolic features in *S. aureus* MTR_BAU_H1 (Figure 6(b)).

## 4. Discussion

MRSA is an important MDR opportunistic pathogen leading to a wide range of infections in both humans [18, 47] and animals [3, 10], resulting in a serious public health and economic burden. While humans are considered its primary reservoir, *S. aureus* can readily cross species barriers and infect new hosts [48]. Here, we performed WGS of a strong biofilm-forming MRSA strain (*S. aureus* MTR_BAU_H1) isolated from food handlers' hand swab samples in Bangladesh to understand the genetic diversity, ST, and distribution of genes associated with antibiotic resistance and virulence. The strain MTR_BAU_H1 was phenotypically resistant to erythromycin, oxacillin, ampicillin, penicillin, and cefoxitin. The strain was further found to harbouring four biofilm-associated genes (e.g., *icaA*, *icaB*, *icaC*, and *icaD*), three ARGs (*mecA*, *blaZ*, and *tet*C), and three enterotoxin-producing virulence genes (e.g., *sea*, *tsst*, and *PVL*). Staphylococcal enterotoxins or virulence genes (e.g., *sea*, *seb*, *tsst*, *PVL*, and others) produced by *S. aureus* are directly associated with staphylococcal food poisoning [49]. This indicates that food handlers in Bangladesh could be an important source of food poisoning (or public threat) due to the carriage of enterotoxin-producing MDR clones of *S. aureus*. The higher prevalence of the *sea* gene in *S. aureus* isolates is not an unusual fact because the isolates with SEA-type toxins cause the most staphylococcal infections and outbreaks, followed by isolates with other staphylococcal enterotoxin-related infections [3, 16].

One of the intriguing findings of this study is that the MTR_BAU_H1 isolate was phenotypically MRSA in nature, indicating a critical threat to food consumers in Bangladesh by limiting the treatment options. Treatment of staphylococcal infections relies mostly on antibiotic therapy; however, it frequently fails due to their resistance to antibiotics. In addition, the presence of MRSA in food handlers' hand raises serious public health implications. MRSA is a growing concern worldwide, and the *mec*A-positive *S. aureus* can be isolated from a wide arsenal of samples [3, 6, 16]. The genotypic detection of *mec*A in biofilm-forming *S. aureus* isolates from food handlers' hand swab samples suggests a serious threat to human health because these resistance gene-containing isolates could easily be transferred to humans via the food supply chain. The MTR_BAU_H1 strain was found positive for beta-lactam resistance gene (*bla*Z), and tetracycline resistance gene (*tet*C). The multidrug resistance of this isolate might be generated by the increased expression of genes that code for multidrug efflux pumps, extruding a wide range of drugs [50]. The detection of ARGs in *S. aureus* isolates from human hand swab samples suggests that these resistance genes might be transferred to other bacteria via horizontal transmission [20]. Genes that encode for antibiotic resistance and superantigens were shared not only between divergent genetic backgrounds (or STs) of *S. aureus* but also between the hosts in which they reside. The WGS also revealed that phenotypically tested MTR_BAU_H1 isolate as MRSA often harbours numerous antibiotic resistance determinants. This result highlights the need to further investigate resistance characteristics beyond *mec*A and *bla*Z resistance in human-associated *S. aureus*, which are often overlooked in many surveillance studies. In addition, such efforts will be instrumental in advancing the One Health concept, focused on the interconnectedness of animal, human, and environmental well-being [1, 51].

We further sought to elucidate the genomic characteristics of *S. aureus* MTR_BAU_H1 isolate sampled from food handlers' hand swab in Bangladesh. The annotated draft genome sequence of *S. aureus* strain MTR_BAU_H1 was 2,764,427 bp length containing 2698 CDS. We found that the *S. aureus* MTR_BAU_H1 strain belongs to ST1930, similar to four MDR STs (three ST96 and one ST154) of *S. aureus* originating from the swab and blood samples of nosocomial and community-associated infections in humans. Remarkably, ST96 isolates have been identified in clinically infected human lesion swab samples from Italy (NCBI accession: NZ_PZRV00000000.1), Denmark (NCBI accession: NZ_OFUU00000000.1), and the United States (NCBI accession: NZ_KI669390.1). These *S. aureus* strains (ST96 and ST154) also harboured different ARGs, including *bla*Z, *mec*A, and *tet*(M) detected in humans and multiple wild and domestic animals [52–54]. The ST1930 S*. aureus* MTR_BAU_H1 was resistant to erythromycin, oxacillin, ampicillin, penicillin, and cefoxitin, which is the distinctive feature of resistance among enterotoxins or virulence (e.g., *sea*, *seb*, *tsst*, and *PVL*)-positive isolates. Although the significance of ST1930 MRSA is not evident, *S. aureus* with CC96 or CC154, to which ST1930 belongs, is revealed to secrete high levels of enterotoxins [55], suggesting relevance to the increased virulence. Therefore, the prevalence of the novel enterotoxin (i.e., *sea*, *seb*, *tsst*, and *PVL*)-positive MRSA clone ST1930 should be carefully monitored in Bangladesh. The strain MTR_BAU_H1 contains seven prophages, and three plasmid replicons (e.g., rep16, rep7c, and rep19). The presence of plasmids and prophage regions in the MTR_BAU_H1 genome might enhance pathogenicity and the capacity of acquiring ARGs, allowing MTR_BAU_H1 to become more virulent and antibiotic-resistant to survive in different environments [56–59]. The predicted plasmids were reported as a potential source of various ARGs that are regulated by three resistance mechanisms: antibiotic inactivation, efflux pump, and antibiotic target alternation [56–59]. One of the recent studies reported that rep plasmid families are diverse among *S. aureus* meat isolates [58]. This study's plasmid types and ARGs identified in the MTR_BAU_H1 genome corroborated well with the available literature worldwide [60]. However, the plasmid replicon types identified in the study isolate were more diverse than the STs. This might be due to the presence of multiple replicon types in the same organism harbouring plasmid. Therefore, for tracking the ARGs and to identify a plasmid outbreak in a food handler's hand swab samples in Bangladesh, plasmid typing is essential. The result of the WGS confirmed the resistance of the isolate to the antibiotics and expanded it to include *mec*A, *bla*Z, aminoglycosides, cephalosporin, chloramphenicol, fluoroquinolones, and tetracycline. Such a result should be considered while planning an effective treatment protocol. The ARGs of *S. aureus* MTR_BAU_H1 revealed that the MTR_BAU_H1 strain isolated from food handlers' hand swabs in Bangladesh is complicated and has a wide range of cross-antibiotic resistance [61].

In order to ascertain the close evolutionary relationship between the MTR_BAU_H1 strain and other MRSA clones of both human and animals, we performed a robust phylogenetic analysis with 26 MRSA genomes sequenced from different continents. The phylogenetic tree established a close evolutionary relationship between the MTR_BAU_H1 genome and other MRSA clones of diverse hosts and demographics. Thus, the MTR_BAU_H1 genome showed a close evolutionary relationship with other MRSA clones of humans sequenced from Japan, Republic of Korea, Brazil, the United States, and the United Kingdom. WGS-based phylogenetic inference of MRSA isolates with reference sequences across time and space enhances our knowledge of the population structure of *S. aureus*, allowing greater precision in describing and defining the different lineages, and offers the potential for an outbreak investigation to determine unambiguously the relatedness of isolates [62–64].

Virulence factors are the degree of pathogenicity of an organism responsible for establishing a disease in the host by combating immunity. A large group of genes conferring virulence factors was found in the genome of the MTR_BAU_H1 strain of the present study. Staphylococci-conserved genes responsible for biofilm formation have a major role in pathogenicity and infection as they contribute to virulence and antimicrobial resistance activity [2]. Another noble aspect of this study is that we identified several genes and/or metabolic pathways correlated with metabolisms of amino acids and derivatives, carbohydrates, protein, cofactors, vitamins, prosthetic groups and pigments, iron, fatty acids, lipids, and isoprenoids. In addition, genes associated with membrane transport, stress response, and regulation and cell signaling were identified in the MTR_BAU_H1 genome. Although much is known about staphylococcal virulence, very little is known about the metabolism of staphylococci. Previous studies on the metabolism and physiology of MRSA have been limited, but the complete genome sequence has allowed for an increased understanding of the basic biology of these species. This study provides novel insights into the genomic characteristics, antimicrobial resistance profiles, and virulence factors of a strong biofilm-producing and MDR *S. aureus* strain, MTR_BAU_H1, isolated from food handlers' hand swabs in Bangladesh. These findings are crucial for informing public health policies aimed at controlling the spread of MRSA through the One Health approach and improving treatment strategies in both human and animal populations. MRSA is a major public concern. Food could be easily contaminated with MRSA, primarily by personnel working or handling food. Good personal hygiene and a clean environment are prerequisites to reducing the occurrence and spread of MRSA. Therefore, we suggest maintaining hygienic measures, including frequent hand washing and wearing gloves, face masks, and protective clothes by food personnel during food handling, preparation, and serving.

However, there are limitations in our study that need to be acknowledged. The *S. aureus* MTR_BAU_H1 isolate, used for WGS in this study, was collected from food handlers' hand swab samples of the Mymensingh district of Bangladesh, resulting in a heavy sampling bias. Moreover, a comparatively low sample size from a single region/district of Bangladesh is another bias. Such bias did not allow us to carry out a more systematic analysis of the host distribution of STs and patterns of gene sharing between human and animal hosts. Future work should therefore include broader surveillance of MRSA in a larger population, including both community and hospital-acquired human samples. Such studies should also include samples from commonly studied domestic animals and wildlife species, especially those that often interface with humans and livestock and/or exist at the junction of urban and natural landscapes.

## 5. Conclusion

The current study is the first of its kind in Bangladesh to give a genomic insight into a strong biofilm-forming MDR *S. aureus* strain, MTR_BAU_H1, isolated from food handlers' hand swabs. The MTR_BAU_H1 strain is resistant to erythromycin, oxacillin, ampicillin, penicillin, and cefoxitin. The MTR_BAU_H1 strain belonging to ST1930 has a wide range of ARGs including *mec*A, *bla*Z, and *tet*C and biofilm-producing (e.g., *icaA*, *icaB*, *icaC*, and *icaD*) and virulence (e.g., *sea*, *tsst*, and *PVL*) genes. Taken together, these results suggest that the *S. aureus* ST1930 strain may represent a public health hazard in Bangladesh. The whole genome of the MTR_BAU_H1 strain can provide a genetic background of virulence, antibiotic resistance, plasmids, and prophages of the MRSA species in Bangladesh. Therefore, monitoring and surveillance of food handlers' hand swabs for MRSA would be beneficial to better define the circulation of these resistant strains and assess the potential risk of resistant infections in community people.

## Figures and Tables

**Table 1:** General features of the *Staphylococcus aureus* strain MTR_BAU_H1 genome.

**Attribute(s)**	**Value**	**% of total ** ^a^
Genome size (bp)	2,764,427	100.00
DNA coding (bp)	2,325,916	84.14
Genome coverage	50×	50
DNA G + C (bp)	1,548,080	56.00
Total contigs	21	
Largest contig (bp)	609,991	22.07
Contig N50 (bp)	279,348	10.10
L50	4	
Total genes	2,746	100.00
Coding sequences (CDSs)	2,698	98.25
Protein coding genes	2,610	95.04
RNA genes	48	1.75
tRNA genes	46	1.67
rRNAs	2 (16S, 23S)	
Pseudogenes	88	3.20
Genes with function prediction	2,480	90.31
Genes assigned to SEED subsystems	2,356	85.80
Number of subsystems	273	
Genes with Pfam domains	2,214	80.63
Genes with signal peptides	318	11.58
Genes with transmembrane helices	902	32.85
CRISPR arrays	2	
Number of plasmids	3 (Inc18, Rep_trans, RepA_N)	
Number of prophages	7	

^a^Total based on either the size of the genome in base pairs (bp) or the total number of genes in the annotated genome.

## Data Availability

The shotgun WGS data of the *S. aureus* strain MTR_BAU_H1 has been deposited at GenBank under accession number JAPJDS010000000, and the assembly reports of the genome are also available at GenBank. The version described in this paper is version JAPJDS000000000.1. The Illumina shotgun reads are available in the National Center for Biotechnology Information (NCBI) Sequence Read Archive (SRA) accession number SRR22312156 under BioProject accession number PRJNA901617.

## Supporting Information

Supporting Information Additional supporting information can be found online in the Supporting Figure S1 CRISPR arrays and associated genes in S. aureus strain MTR_BAU_H1 genome. Table S1 Virulence factor genes (VFGs) identified in *Staphylococcus aureus* strain MTR_BAU_H1 genome.

## References

[1] Ballah F. M., Islam M. S., Rana M. L. (2022). Phenotypic and genotypic detection of biofilm-forming *Staphylococcus aureus* from different food sources in Bangladesh. *Biology*.

[2] Hoque M. N., Istiaq A., Clement R. A. (2020). Insights into the resistome of bovine clinical mastitis microbiome, a key factor in disease complication. *Frontiers in Microbiology*.

[3] Hoque M., Das Z., Rahman A., Haider M., Islam M. (2018). Molecular characterization of *Staphylococcus aureus* strains in bovine mastitis milk in Bangladesh. *International Journal of Veterinary Science and Medicine*.

[4] Hoque M. N., Faisal G. M., Chowdhury F. R., Haque A., Islam T. (2022). The urgency of wider adoption of one health approach for the prevention of a future pandemic. *International Journal of One Health*.

[5] Klein E. Y., Sun L., Smith D. L., Laxminarayan R. (2013). The changing epidemiology of methicillin-resistant *Staphylococcus aureus* in the United States: a national observational study. *American Journal of Epidemiology*.

[6] Bruce S. A., Smith J. T., Mydosh J. L. (2022). Shared antibiotic resistance and virulence genes in *Staphylococcus aureus* from diverse animal hosts. *Scientific Reports*.

[7] Tong S. Y., Davis J. S., Eichenberger E., Holland T. L., Fowler V. G. (2015). *Staphylococcus aureus* infections: epidemiology, pathophysiology, clinical manifestations, and management. *Clinical Microbiology Reviews*.

[8] Schuler F., Barth P. J., Niemann S., Schaumburg F. (2021). A narrative review on the role of *Staphylococcus aureus* bacteriuria in S. aureus bacteremia. *Open Forum Infectious Diseases*.

[9] Hoque M. N., Istiaq A., Rahman M. S. (2020). Microbiome dynamics and genomic determinants of bovine mastitis. *Genomics*.

[10] Hoque M. N., Talukder A. K., Saha O. (2022). Antibiogram and virulence profiling reveals multidrug resistant *Staphylococcus aureus* as the predominant aetiology of subclinical mastitis in riverine buffaloes. *Veterinary Medicine and Science*.

[11] Fitzgerald J. R. (2012). Livestock-associated *Staphylococcus aureus*: origin, evolution and public health threat. *Trends in Microbiology*.

[12] Fooladi A. A., Ashrafi E., Tazandareh S. G. (2015). The distribution of pathogenic and toxigenic genes among MRSA and MSSA clinical isolates. *Microbial Pathogenesis*.

[13] Piechota M., Kot B., Frankowska-Maciejewska A., Grużewska A., Woźniak-Kosek A. (2018). Biofilm formation by methicillin-resistant and methicillin-sensitive *Staphylococcus aureus* strains from hospitalized patients in Poland. *BioMed Research International*.

[14] Chung H. Y., Kim Y.-T., Kwon J.-G. (2021). Molecular interaction between methicillin-resistant *Staphylococcus aureus* (MRSA) and chicken breast reveals enhancement of pathogenesis and toxicity for food-borne outbreak. *Food Microbiology*.

[15] da Silva A. C., Rodrigues M. X., Silva N. C. C. (2020). Methicillin-resistant *Staphylococcus aureus* in food and the prevalence in Brazil: a review. *Brazilian Journal of Microbiology*.

[16] Ballah F. M., Islam M. S., Rana M. L. (2022). Virulence determinants and methicillin resistance in biofilm-forming *Staphylococcus aureus* from various food sources in Bangladesh. *Antibiotics*.

[17] Prenafeta A., Sitjà M., Holmes M. A., Paterson G. K. (2014). Short communication: biofilm production characterization of mecA and mecC methicillin-resistant *Staphylococcus aureus* isolated from bovine milk in Great Britain. *Journal of Dairy Science*.

[18] Algammal A. M., Hetta H. F., Elkelish A. (2020). Methicillin-resistant *Staphylococcus aureus* (MRSA): one health perspective approach to the bacterium epidemiology, virulence factors, antibiotic-resistance, and zoonotic impact. *Infection and Drug Resistance*.

[19] Al Amin M., Hoque M. N., Siddiki A. Z., Saha S., Kamal M. M. (2020). Antimicrobial resistance situation in animal health of Bangladesh. *Veterinary World*.

[20] Gajdács M., Zsoldiné Urbán E. (2019). Epidemiology and resistance trends of *Staphylococcus aureus* isolated from vaginal samples: a 10-year retrospective study in Hungary. *Acta Dermatovenerologica Alpina, Pannonica et Adriatica*.

[21] Cerca N., Jefferson K. K., Maira-Litrán T. (2007). Molecular basis for preferential protective efficacy of antibodies directed to the poorly acetylated form of staphylococcal poly-*N*-acetyl-*β*-(1-6)-glucosamine. *Infection and Immunity*.

[22] Hoque M. N., Jahan M. I., Hossain M. A., Sultana M. (2022). Genomic diversity and molecular epidemiology of a multidrug-resistant Pseudomonas aeruginosa DMC30b isolated from a hospitalized burn patient in Bangladesh. *Journal of Global Antimicrobial Resistance*.

[23] CLSI *Performance Standards for Antimicrobial Susceptibility Testing*.

[24] Bolger A. M., Lohse M., Usadel B. (2014). Trimmomatic: a flexible trimmer for Illumina sequence data. *Bioinformatics*.

[25] Bankevich A., Nurk S., Antipov D. (2012). SPAdes: a new genome assembly algorithm and its applications to single-cell sequencing. *Journal of Computational Biology*.

[26] Gurevich A., Saveliev V., Vyahhi N., Tesler G. (2013). QUAST: quality assessment tool for genome assemblies. *Bioinformatics*.

[27] Singh S., Pulusu C. P., Pathak A., Pradeep B. E., Prasad K. N. (2021). Complete genome sequence of an extensively drug-resistant Pseudomonas aeruginosa ST773 clinical isolate from North India. *Journal of Global Antimicrobial Resistance*.

[28] Akter T., Haque M. N., Ehsan R. (2023). Virulence and antibiotic-resistance genes in Enterococcus faecalis associated with streptococcosis disease in fish. *Scientific Reports*.

[29] Katoh K., Asimenos G., Toh H. (2009). Multiple alignment of DNA sequences with MAFFT. *Methods in Molecular Biology*.

[30] Letunic I., Bork P. (2016). Interactive tree of life (iTOL) v3: an online tool for the display and annotation of phylogenetic and other trees. *Nucleic Acids Research*.

[31] Grant J. R., Stothard P. (2008). The CGView server: a comparative genomics tool for circular genomes. *Nucleic Acids Research*.

[32] Carattoli A., Zankari E., García-Fernández A. (2014). In silico detection and typing of plasmids using PlasmidFinder and plasmid multilocus sequence typing. *Antimicrobial Agents and Chemotherapy*.

[33] Arndt D., Grant J. R., Marcu A. (2016). PHASTER: a better, faster version of the PHAST phage search tool. *Nucleic Acids Research*.

[34] Feng Y., Zou S., Chen H., Yu Y., Ruan Z. (2021). BacWGSTdb 2.0: a one-stop repository for bacterial whole-genome sequence typing and source tracking. *Nucleic Acids Research*.

[35] Feldgarden M., Brover V., Haft D. H. (2019). Validating the AMRFinder tool and resistance gene database by using antimicrobial resistance genotype-phenotype correlations in a collection of isolates. *Antimicrobial Agents and Chemotherapy*.

[36] Alcock B. P., Raphenya A. R., Lau T. T. (2020). CARD 2020: antibiotic resistome surveillance with the comprehensive antibiotic resistance database. *Nucleic Acids Research*.

[37] Gupta S. K., Padmanabhan B. R., Diene S. M. (2014). ARG-ANNOT, a new bioinformatic tool to discover antibiotic resistance genes in bacterial genomes. *Antimicrobial Agents and Chemotherapy*.

[38] Bortolaia V., Kaas R. S., Ruppe E. (2020). ResFinder 4.0 for predictions of phenotypes from genotypes. *Journal of Antimicrobial Chemotherapy*.

[39] Doster E., Lakin S. M., Dean C. J. (2020). MEGARes 2.0: a database for classification of antimicrobial drug, biocide and metal resistance determinants in metagenomic sequence data. *Nucleic Acids Research*.

[40] Liu B., Zheng D., Jin Q., Chen L., Yang J. (2019). VFDB 2019: a comparative pathogenomic platform with an interactive web interface. *Nucleic Acids Research*.

[41] Ievy S., Hoque M. N., Islam M. S. (2022). Genomic characteristics, virulence, and antimicrobial resistance in avian pathogenic Escherichia coli MTR_BAU02 strain isolated from layer farm in Bangladesh. *Journal of Global Antimicrobial Resistance*.

[42] Hoque M. N., Moyna Z., Faisal G. M., Das Z. C. (2023). Whole-genome sequence of the multidrug-resistant Staphylococcus warneri strain G1M1F, isolated from mice with mastitis. *Microbiology Resource Announcements*.

[43] Hoque M. N., Moyna Z., Faisal G. M., Das Z. C., Islam T. (2023). Whole-genome sequence of multidrug-resistant Klebsiella pneumoniae MNH_G2C5, isolated from bovine clinical mastitis milk. *Microbiology Resource Announcements*.

[44] Aziz R. K., Bartels D., Best A. A. (2008). The RAST server: rapid annotations using subsystems technology. *BMC Genomics*.

[45] Moriya Y., Itoh M., Okuda S., Yoshizawa A. C., Kanehisa M. (2007). KAAS: an automatic genome annotation and pathway reconstruction server. *Nucleic Acids Research*.

[46] Saha O., Rakhi N. N., Hoque M. N., Sultana M., Hossain M. A. (2021). Genome-wide genetic marker analysis and genotyping of Escherichia fergusonii strain OTSVEF-60. *Brazilian Journal of Microbiology*.

[47] Ngoi S. T., Niek W. K., Lee Y. W., AbuBakar S., Teh C. S. J. (2021). Genomic analysis revealed a novel genotype of methicillin-susceptible *Staphylococcus aureus* isolated from a fatal sepsis case in dengue patient. *Scientific Reports*.

[48] Richardson E. J., Bacigalupe R., Harrison E. M. (2018). Gene exchange drives the ecological success of a multi-host bacterial pathogen. *Nature Ecology & Evolution*.

[49] Fisher E. L., Otto M., Cheung G. Y. (2018). Basis of virulence in enterotoxin-mediated staphylococcal food poisoning. *Frontiers in Microbiology*.

[50] Chambers H. F., DeLeo F. R. (2009). Waves of resistance: *Staphylococcus aureus* in the antibiotic era. *Nature Reviews Microbiology*.

[51] Zinsstag J., Schelling E., Waltner-Toews D., Tanner M. (2011). From “one medicine” to “one health” and systemic approaches to health and well-being. *Preventive Veterinary Medicine*.

[52] Nair R., Hanson B. M., Kondratowicz K. (2013). Antimicrobial resistance and molecular epidemiology of *Staphylococcus aureus* from Ulaanbaatar, Mongolia. *PeerJ*.

[53] Penadés M., Viana D., García-Quirós A. (2020). Differences in virulence between the two more prevalent *Staphylococcus aureus* clonal complexes in rabbitries (CC121 and CC96) using an experimental model of mammary gland infection. *Veterinary Research*.

[54] Rossney A. S., Shore A. C., Morgan P. M., Fitzgibbon M. M., O'Connell B., Coleman D. C. (2007). The emergence and importation of diverse genotypes of methicillin-resistant *Staphylococcus aureus* (MRSA) harboring the Panton-Valentine leukocidin gene (pvl) reveal that pvl is a poor marker for community-acquired MRSA strains in Ireland. *Journal of Clinical Microbiology*.

[55] Monecke S., Müller E., Büchler J., Stieber B., Ehricht R. (2014). *Staphylococcus aureus* in vitro secretion of alpha toxin (hla) correlates with the affiliation to clonal complexes. *PloS One*.

[56] Jans C., Wambui J., Stevens M. J., Tasara T. (2022). Comparative genomics of dairy-associated *Staphylococcus aureus* from selected sub-Saharan African regions reveals milk as reservoir for human-and animal-derived strains and identifies a putative animal-related clade with presumptive novel siderophore. *Frontiers in Microbiology*.

[57] Naorem R. S., Blom J., Fekete C. (2021). Genome-wide comparison of four MRSA clinical isolates from Germany and Hungary. *PeerJ*.

[58] Neyaz L., Rajagopal N., Wells H., Fakhr M. K. (2020). Molecular characterization of *Staphylococcus aureus* plasmids associated with strains isolated from various retail meats. *Frontiers in Microbiology*.

[59] Stanczak-Mrozek K. I., Manne A., Knight G. M., Gould K., Witney A. A., Lindsay J. A. (2015). Within-host diversity of MRSA antimicrobial resistances. *Journal of Antimicrobial Chemotherapy*.

[60] Ragupathi N. K. D., Bakthavatchalam Y. D., Mathur P. (2019). Plasmid profiles among some ESKAPE pathogens in a tertiary care centre in South India. *The Indian Journal of Medical Research*.

[61] Ali M. S., Isa N. M., Abedelrhman F. M. (2019). Genomic analysis of methicillin-resistant *Staphylococcus aureus* strain SO-1977 from Sudan. *BMC Microbiology*.

[62] Harris S. R., Cartwright E. J., Török M. E. (2013). Whole-genome sequencing for analysis of an outbreak of meticillin-resistant *Staphylococcus aureus*: a descriptive study. *The Lancet Infectious Diseases*.

[63] Humphreys H., Coleman D. (2019). Contribution of whole-genome sequencing to understanding of the epidemiology and control of meticillin-resistant *Staphylococcus aureus*. *Journal of Hospital Infection*.

[64] Price J., Didelot X., Crook D., Llewelyn M., Paul J. (2013). Whole genome sequencing in the prevention and control of *Staphylococcus aureus* infection. *Journal of Hospital Infection*.

