# Supplementary material for: Genomic Insights of a Methicillin-Resistant Biofilm-Producing Staphylococcus aureus Strain Isolated From Food Handlers
Source: Biomed Res Int. 2024 Jul 20;2024:5516117. doi: 10.1155/2024/5516117 (PMC11283335; doi:10.1155/2024/5516117)
Supplement: Supporting Information — Additional supporting information can be found online in the Supporting Figure S1 CRISPR arrays and associated genes in S. aureus strain MTR_BAU_H1 genome. Table S1 Virulence factor genes (VFGs) identified in Staphylococcus aureus strain MTR_BAU_H1 genome. [file 5516117.f1.pdf]

# Supplementary Figure

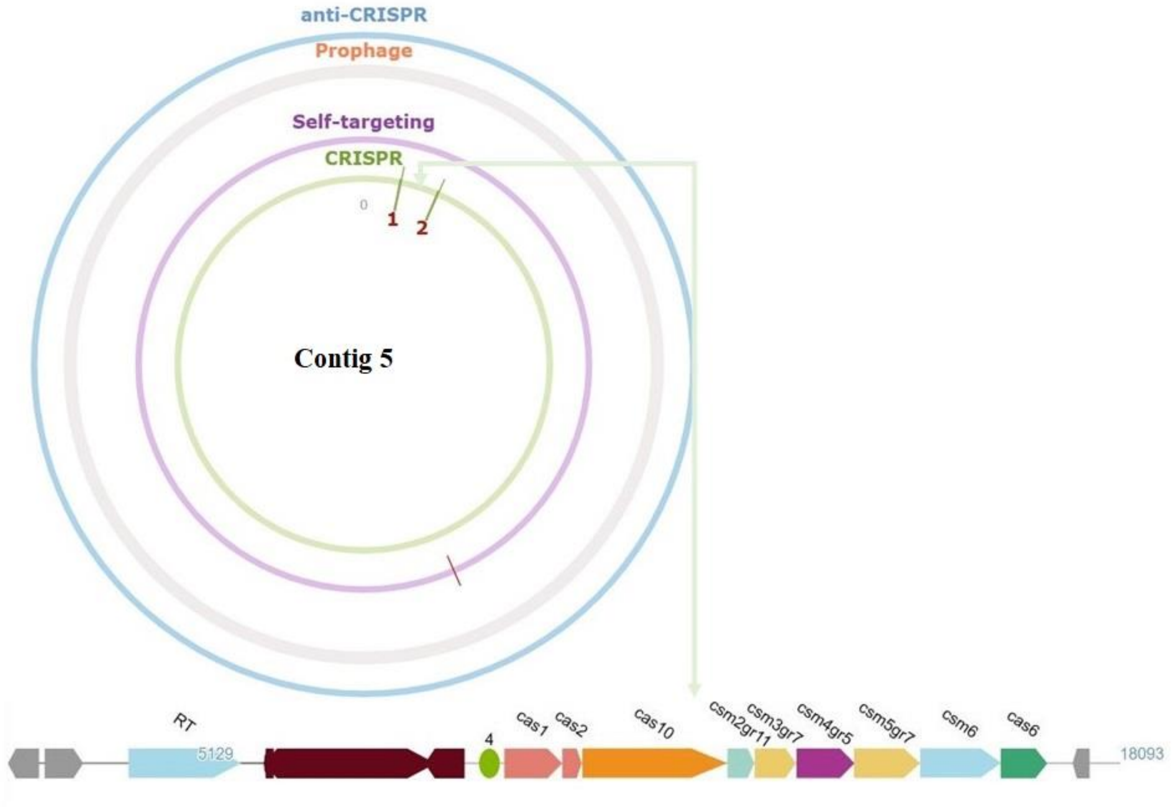


**Fig. S1.** CRISPR arrays and associated genes in *S. aureus* strain MTR_BAU_H1 genome.

# Supplementary Table

**Table S1:** Virulence factor genes (VFGs) identified in Staphylococcus aureus strain MTR_BAU_H1 genome.

| VFclass | Virulence factors | Related genes | % Identity |
|---|---|---|---|
| Adherence | Autolysin | atl | 100 |
| | Cell wall associated fibronectin binding protein | ebh | 100 |
| | Clumping factor A | clfA | 97.57 |
| | Clumping factor B | clfB | 92.68 |
| | Collagen adhesion | cna | 98.93 |
| | Elastin binding protein | ebp | 97.95 |
| | Extracellular adherence protein/MHC analogous protein | eap/map | 91.23 |
| | Fibrinogen binding protein | efb | 97.95 |
| | Fibronectin binding proteins | fnbA | 97.95 |
| | | fnbB | 97.95 |
| | Intercellular adhesin | icaA | 99.6 |
| | | icaB | 99.54 |
| | | icaC | 98.39 |
| | | icaD | 100 |
| | | icaR | 99.82 |
| | Ser-Asp rich fibrinogen-binding proteins | sdrC | 92.28 |
| | | sdrD | 100 |

| | | | |
|---|---|---|---|
| | | sdrE | 94.82 |
| | | sdrF | 99.54 |
| | | sdrG | 98.39 |
| | | sdrH | 100 |
| | Staphylococcal protein A | spa | 99.82 |
| Enzymatic activity | Cysteine protease | sspB | 99.07 |
| | | sspC | 99.7 |
| | Hyaluronate lyase | hysA | 93.7 |
| | Lipase | geh | 99.33 |
| | | lip | 99.66 |
| | Serine V8 protease | sspA | 96.83 |
| | Serine protease | splA | 99.82 |
| | | splB | 99.07 |
| | | splC | 99.7 |
| | | splD | 94.7 |
| | | splE | 99.33 |
| | | splF | 99.66 |
| | Staphylocoagulase | coa | 96.83 |
| | Staphylokinase | sak | 99.39 |
| | Thermonuclease | nuc | 100 |
| Immune evasion | AdsA | adsA | 97.15 |
| | CHIPS | chp | 100 |
| | Capsule | Undetermined | 100 |

| | | | |
|---|---|---|---|
| | SCIN | scn | 100 |
| | Sbi | sbi | 99.01 |
| Secretion system | Type VII secretion system | esaA | 98.84 |
| | | esaB | 98.77 |
| | | esaD | 99.01 |
| | | esaE | 98.84 |
| | | esaG | 98.77 |
| | | essA | 98.47 |
| | | essB | 98.5 |
| | | essC | 93.51 |
| | | esxA | 100 |
| | | esxB | 99.01 |
| | | esxC | 98.84 |
| | | esxD | 98.77 |
| Toxin | Alpha hemolysin | hly/hla | 99.69 |
| | Beta hemolysin | hlb | 99.64 |
| | Delta hemolysin | hld | 100 |
| | Enterotoxin A | sea | 98.71 |
| | Enterotoxin B | seb | 100 |
| | Enterotoxin C | sec | 99.5 |
| | Enterotoxin D | sed | 100 |
| | Enterotoxin E | see | 99.01 |
| | Enterotoxin G | seg | 98.84 |
| | Enterotoxin H | seh | 98.77 |

| | | | |
|---|---|---|---|
| | Enterotoxin I | sei | 99.69 |
| | Enterotoxin J | sej | 99.64 |
| | Enterotoxin Yent1 | yent1 | 100 |
| | Enterotoxin Yent2 | yent2 | 99.01 |
| | Enterotoxin-like K | selk | 98.84 |
| | Enterotoxin-like L | sell | 98.77 |
| | Enterotoxin-like M | selm | 99.69 |
| | Enterotoxin-like N | seln | 99.64 |
| | Enterotoxin-like O | selo | 100 |
| | Enterotoxin-like P | selp | 99.01 |
| | Enterotoxin-like Q | selq | 98.84 |
| | Enterotoxin-like R | selr | 98.77 |
| | Enterotoxin-like U | selu | 99.69 |
| | Exfoliative toxin type A | eta | 99.64 |
| | Exfoliative toxin type B | etb | 99.01 |
| | Exfoliative toxin type C | etc | 98.84 |
| | Exfoliative toxin type D | etd | 98.77 |
| | Exotoxin | set10 | 99.69 |
| | | set11 | 99.64 |
| | | set12 | 100 |
| | | set13 | 99.01 |
| | | set14 | 98.84 |
| | | set15 | 98.77 |
| | | set16 | 99.69 |

| | | | |
|---|---|---|---|
| | | set17 | 99.64 |
| | | set18 | 100 |
| | | set19 | 99.01 |
| | | set1 | 98.84 |
| | | set20 | 98.77 |
| | | set21 | 99.69 |
| | | set22 | 99.64 |
| | | set23 | 99.01 |
| | | set24 | 98.84 |
| | | set25 | 98.77 |
| | | set26 | 99.69 |
| | | set2 | 99.64 |
| | | set30 | 100 |
| | | set31 | 99.01 |
| | | set32 | 99.01 |
| | | set33 | 98.84 |
| | | set34 | 98.77 |
| | | set35 | 99.69 |
| | | set36 | 99.64 |
| | | set37 | 99.01 |
| | | set38 | 98.84 |
| | | set39 | 99.01 |
| | | set3 | 98.84 |
| | | set40 | 98.77 |

| | | | |
|---|---|---|---|
| | | set4 | 99.69 |
| | | set5 | 99.64 |
| | | set6 | 99.01 |
| | | set7 | 98.84 |
| | | set8 | 98.77 |
| | | set9 | 99.69 |
| | Gamma hemolysin | hlgA | 99.64 |
| | | hlgB | 100 |
| | | hlgC | 99.01 |
| | Leukocidin M | lukF-like | 99.69 |
| | | lukM | 99.64 |
| | Leukotoxin D | lukD | 100 |
| | Leukotoxin E | lukE | 99.01 |
| | Panton-Valentine leukocidin | lukF-PV | 99.69 |
| | | lukS-PV | 99.64 |
| | Toxic shock syndrome toxin | tsst | 100 |
